# Characterization of body weight and composition changes during the sophomore year of college

**DOI:** 10.1186/1472-6874-7-21

**Published:** 2007-11-20

**Authors:** Holly R Hull, Michelle L Morrow, Mary K Dinger, Jennifer L Han, David A Fields

**Affiliations:** 1 Department of Medicine, Obesity Research Center, St. Luke's-Roosevelt Hospital, New York, NY, USA; 2 University of Oklahoma Health Sciences Center, Oklahoma City, OK, USA; 3 Department of Health and Exercise Science, University of Oklahoma, Norman, OK, USA; 4 Department of Pediatrics, University of Oklahoma Health Science Center, Oklahoma City, OK, USA; 5 Children's Medical Research Institute's Metabolic Research Center, Oklahoma City, OK, USA

## Abstract

**Background:**

Years spent in college represents a critical time for obesity development though little information is known regarding how body weight and composition changes beyond the first year of college. The aim of this study was to investigate changes in body weight and composition and the factors influencing those changes among sophomore females.

**Methods:**

Body composition by dual energy X-ray absorptiometry was obtained in participants beginning during their freshman year and continued through their sophomore year.

**Results:**

No difference was observed between sophomore year fall and spring visits for body weight (60.4 versus 60.6 kg) or fat mass (19.3 versus 18.7 kg). However, a significant (*P *≤ 0.05) decrease was observed for body fat (31.9 versus 30.9 %fat) and a significant increase was observed for fat-free mass (37.7 versus 38.4 kg). Participants living off campus significantly (*P *≤ 0.05) declined in body fat (33.0 versus 31.0 %fat) and fat mass (19.4 versus 18.2 kg) and increased in fat-free mass (36.1 versus 37.2 kg) with no differences in those living on campus.

**Conclusion:**

No change in body weight was observed in females during their sophomore year. However, an increase in fat-free mass accompanied with a decrease in fat mass resulted in a decrease in body fat. Participants living off campus had favorable changes in their body composition by means of decreasing %fat and fat mass while increasing fat-free mass. Participants living on campus did not demonstrate these favorable changes.

## Background

It is widely accepted that substantial weight gain occurs during the first year of college therefore the phrase 'Freshman 15' is often echoed on college campuses. However no study has actually shown the purported 15 pound increase, instead an average of 1.9 kg (4.2 pound) has been reported between all studies completed [1-11]. Possibly more important and warranting further inquiry than weight gained in solely the first year of college is whether weight gained is lost, maintained or if additional weight is added. This information could provide a crucial model of slow weight gain thought to be at the root of our current obesity epidemic.

Increases in obesity levels are commonly attributed to small prolonged increases in energy intake resulting in a gradual consistent yearly weight gain. In young adults this has been estimated to be approximately 0.2 to 0.8 kg per year [12]. At this time, only two studies [5,10] have attempted to follow students beyond their first year of college to determine if weight gained during the freshman year is carried into and through the subsequent sophomore year. Therefore, this population provides a unique opportunity for investigation so that critical time points of obesity development can be better understood.

Another aspect possibly impacting weight gain in female college students yet to be explored is the impact of living on campus versus living off campus. Traditionally, during the first year of college many students reside on campus. However after the freshman year, students are more likely to move off campus. At this time, no study has followed female students during their freshman year and through their sophomore year to determine if living on campus versus living off campus affects body weight and composition.

Data regarding weight change during the freshman year range from 0.7 kg to 3.2 kg [1-11]. The inconsistencies are partly due to the duration of the study, method of weight measurement, and small sample size. A major limitation of past studies is that percent fat (%fat) has only been determined in a few studies [2-4,9,11]. Furthermore, in studies assessing body composition, either skinfolds measurements or bioelectrical impedance was used, thus the usefulness of the data is debatable. To our knowledge only one study examining freshman year changes in body weight and composition has used a criterion method, dual energy X-ray absorptiometry (DXA), to determine %fat and body composition [9].

This study is unique in that prior to this study, only two studies have attempted to examine weight gain through the sophomore year of college, with none quantifying body composition [5,10] and none exploring the possible impact of living on campus versus living off campus. Data presented in this manuscript are part of an original cohort of females initially recruited during their freshman year of college (fall 2004) to participate in a longitudinal study tracking changes in both body weight and composition during the formative college years at a large Midwestern university (University of Oklahoma) [9]. The purposes of this manuscript were three fold: 1) characterize and quantify changes in body weight and composition during the sophomore year of college, 2) compare absolute differences in body weight and composition (fat mass and fat-free mass) between the freshman and sophomore years of college, and 3) compare absolute differences and changes in body weight and composition in those living on campus to those living off campus during their sophomore year.

## Methods

### Participants

Participants were enrolled as freshman during the fall 2004 semester with the methods described elsewhere [9]. Briefly, the inclusion criteria included females enrolled as full time students at the University of Oklahoma (Norman campus) for their academic sophomore year (fall 2005 – spring 2006) and completed the 'Freshman Fifteen' study for their academic freshman Year (fall 2004 – spring 2005). Exclusion criteria included the following: not completing both the fall 2004 and spring 2005 visits for the prior freshman year, pregnant or planning to become pregnant during the study period, participation in any of the university's intercollegiate or club athletic teams, having a metabolic disease that affects body weight and body distribution (e.g., Cushing's Syndrome), and taking medication or drugs known to impact body weight and distribution (e.g., steroids, growth hormone, ephedrine, and nicotine).

One hundred seventy-one females completed the initial fall semester visit during their freshman year of college (fall 2004 semester) with 48 participants completing all four study visits (freshman and sophomore fall and spring visits). Consequently study results presented in this manuscript only include participants that completed all study visits during their freshman and sophomore years. The demographic characteristics of the study completers are presented in Table 1. During the sophomore year fall visit, participants were 19 years old, living primarily off campus, and were predominately white. The freshman year body weight and composition data have been reported elsewhere [9].

**Table 1:** Descriptive characteristics of study completers for the sophomore year (*N *= 48)

**Variables**	**Fall 2005**
Age (yrs)	19.2 ± 0.4
Height (cm)	165.7 ± 6.1
Weight (kg)	60.4 ± 8.2
BMI (kg/m^2^)	22.5 ± 3.8

Housing: n (%)	
On Campus (Dormitory or Sorority Housing)	18 (38%)
Other College Housing	4 (8%)
Off Campus	25 (52%)
Parent's Home	1 (2%)

Race: n (%)	
American Indian	3 (6%)
African American	1 (2%)
Hispanic	0 (0%)
Caucasian	43 (90%)
Other	1 (2%)

Mean ± standard deviation

### Study Design

The study used a prospective longitudinal, one-group research design with the primary outcomes variables being body weight and composition. Data were collected during the first 6 weeks of the fall 2005 semester and the last 6 weeks of the spring 2006 semester during the sophomore year. Participants had previously completed the same testing procedure during their freshman year.

### Measures

A balance beam scale and stadiometer (Detecto Manual Physician, Webb City, MO) were used to measure weight and height with both assessed with shoes and all heavy clothing (e.g., jackets, sweaters, and belts) removed. Participants completed a questionnaire to assess housing status as either on campus (college dormitory, sorority house or other on campus college housing) or off campus (house, rental property, or parent's home).

Body composition, specifically %fat, fat mass, and fat-free mass, was assed by dual energy x-ray absorptiometry (DXA) (DPX-IQ software version 4.7b, Lunar Corporation, Madison, WI) with calibration occurring at the beginning of each day prior to testing. Participants were instructed to lie supine on the DXA table and centered within the scanning field. Straps were placed around the ankles and just below the knees to keep participants in the correct position. Scan speed was determined by measuring anterior posterior thickness at the midsection. The day-to-day coefficient of variation for the estimation of percent fat in our laboratory is 1%.

Recruitment methods have been described elsewhere and are briefly described here [9]. Participants in the original Freshman Fifteen study were contacted via email or telephone and invited to continue participating throughout their college careers. All study visits occurred within the first 6 weeks of the fall semester and within the last 6 weeks of the spring semester. Instructions were given to fast for 6 hours and refrain from exercise for 24 hours prior to their visit. At the initial baseline visit during the fall of the freshman year, eligibility was confirmed and the participant completed an informed consent and HIPAA form which had been approved by the Institutional Review Board at the University of Oklahoma. Upon returning during the fall of their sophomore year eligibility was confirmed again. The average number of days between the fall and spring visits during the sophomore year was 205 days, with a range of 187 to 225 days. Participants received a printout with their body weight and composition at the end of each testing session.

### Data Analysis

Means and standard deviations were calculated for all outcome variables. Changes in the outcome variables between visits (i.e. body weight and body composition) were evaluated by paired t-tests as was the change in body weight and body composition between the freshman year and the sophomore years. Participants were then characterized by housing status; either on campus (college dormitory, sorority house or other on campus college housing) or off campus (house, rental property, or parent's home) and the changes in outcome variables between visits were again evaluated by paired t-tests. Statistical significance was set at *P *≤ 0.05.

## Results

Body weight did not significantly change between the fall and spring sophomore visits; however, a significant (*P *≤ 0.05) decrease in %fat and significant increase in fat-free mass between the fall and spring visits was observed (Table 2).

**Table 2:** Body weight and body composition during the sophomore year

	Sophomore Year (N = 48)		
	Fall 2005	Spring 2006	Change in (Spring to Fall)

Body Weight (kg)	60.4 ± 8.2	60.6 ± 8.7	0.2
Body Fat (%)	31.9 ± 5.8	30.9 ± 5.3	-1.0*
Fat Mass (kg)	19.3 ± 5.6	18.7 ± 5.4	-0.6
Fat-Free Mass (kg)	37.7 ± 4.0	38.4 ± 4.1	0.7*

Mean ± standard deviation

*Significantly different from the fall 2005 at *P *< 0.05

**Figure 1:**
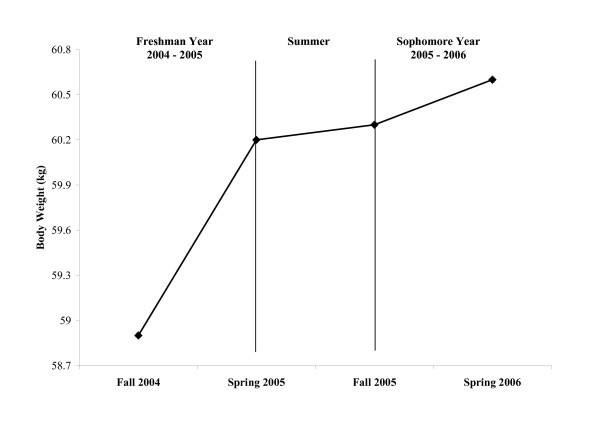
Depiction of the mean values for body weight at each visit (N = 48).

When comparing the absolute change between the freshman and sophomore years, significant (*P *≤ 0.05) differences were found for body weight, %fat, and fat mass (Table 3 and Figure 1). The absolute change for body weight was smaller in the sophomore year versus the freshman year while %fat and total fat mass actually decreased during the sophomore year relative to increasing during the freshman year (Table 3).

**Table 3:** Comparison of changes in body weight and body composition between the freshman and sophomore academic years

	Change in Freshman Year (Fall 2004 to Spring 2005)	Change in Sophomore Year (Fall 2005 to Spring 2006)
Body Weight (kg)	1.2	0.2*
Body Fat (%)	0.7	-1.0*
Fat Mass (kg)	0.8	-0.6*
Fat-Free Mass (kg)	0.5	0.7

*Change significantly different from freshman year at *P *< 0.05

The results of changes in body weight and composition based on housing status are presented in Table 4. Those living on campus had no change in body weight or body composition variables between the fall and spring visits; however, those living off campus declined significantly (*P *≤ 0.05) in %fat and fat mass while increasing significantly in fat-free mass between the fall 2005 and spring 2006 visits.

**Table 4:** Comparison of changes in body weight and body composition during the sophomore year by housing status (on campus versus off campus)

	On Campus (N = 22)			Off Campus (N = 26)		
	Fall 2005	Spring 2006	Change in	Fall 2005	Spring 2006	Change in

Body Weight (kg)	62.3 ± 9.1	62.7 ± 9.6	0.4	58.8 ± 7.2	58.8 ± 7.6	0
Body Fat (%)	30.6 ± 6.0	30.7 ± 5.0	0.1	33.0 ± 5.7	31.0 ± 5.7^†^	-2.0*
Fat Mass (kg)	19.1 ± 6.3	19.3 ± 5.9	0.2	19.4 ± 5.0	18.2 ± 5.0^†^	-1.2*
Fat-Free Mass (kg)	39.5 ± 4.1	39.9 ± 4.1	0.4	36.1 ± 3.3^§^	37.2 ± 3.7^†^	1.1*

Δ Spring to fall

*Change significantly different from on campus group at *P *< 0.05

^†^Significantly different from fall 2005 at *P *< 0.05

^§^Significantly different from on campus fall 2005 *P *< 0.05

## Discussion

This study examined changes in body weight and composition in females during their sophomore year of college and the impact of living on and off campus. Furthermore, it explored potential absolute differences in body weight and composition (fat mass and fat-free mass) between the freshman and sophomore years of college. This is important because to date only a paucity of literature exists that have examined changes in body composition through the sophomore year of college with no data we are aware of that have investigated the role of living arrangement on this relationship. Our major finding is that %fat significantly decreased while fat-free mass significantly increased over the course of the sophomore year and it appeared that the sophomore year resulted in total body fat mass returning to values observed at the beginning of the freshman year. This is evident in fat mass significantly decreasing (0.6 kg) during the sophomore year, while significantly (0.8 kg) increasing during the freshman year.

### Sophomore year findings

Only two studies to date have examined body weight during the sophomore year of college with both showing increases ranging from 0.3 to 0.6 kg in both males and females, of which are larger than observed (0.2 kg) in this study [5,10]. Distinct differences exist between our study population and the study population of Racette et al., possibly explaining the large difference in findings. Our study tested only females whereas the Racette et al. included both males and females. Thus, their 0.6 kg increase found during the sophomore year may be partially attributed to the inclusion of male participants. Furthermore, none of these studies attempted to assess body composition during the sophomore year. Aside from our study, only four studies [2,5,10,11] have quantified body composition (using bioelectric impedance or skinfolds) during the freshman year with results ranging from a 0.4% loss to a 2.1 % gain. Our results showed an increase of 1.0 %fat units for the sophomore year.

### Sophomore versus freshman year findings

Of interest is the difference in results for the absolute change and the rate of change (not reported) between the sophomore and freshman year for the study variables (body weight, %fat, and fat mass) (reported in Table 3). A greater increase in body weight during the freshman year (rate of change 0.18 kg/month) was found when compared to the sophomore year (rate of change 0.02 kg/month) where the total weight gained and the rate of weight gain per month slowed dramatically. Perhaps of greater interest though, are the contrasting results between the absolute differences and changes in %fat and fat mass. While the rates of change per month for %fat and fat mass are comparable between the freshman and sophomore years, the directions are exactly opposite. Percent fat and fat mass increased during the freshman year (0.7 %fat units and 0.8 kg) while they decreased during the sophomore year (-1.0 %fat units and -0.6 kg) even though the absolute difference in fat-free mass and the rate of change in fat free-mass between the freshman and sophomore years were similar. This indicates that the loss of weight during the sophomore year was a loss of fat mass with maintenance of fat-free mass.

### On campus versus off-campus findings

In an attempt to gain a greater appreciation and insight into the impact of the on campus environment and the role on body weight and composition we stratified participants based on where students lived during their sophomore year: either on campus (college dormitory, sorority house, or other on campus housing) or off campus (house, rental property, or parent's home). Interestingly, those living off campus had more favorable changes in body weight and composition, this is to say they lost body fat and fat mass while gaining fat-free mass with no significant changes in body weight (Table 4). This is contradictory to those living on campus who gained body weight, with slight gains in %fat, fat mass and fat-free mass. This study did not directly assess differences in eating patterns of these two groups, but those living on campus by university policy must have a meal plan. There are several meal plans with each plan having a mixture of a set number of meals and meal points. Meal points can only be spent at any university eating establishment (i.e. restaurants and cafés). Though speculative, this may demonstrate that participants that live on campus have easier access to food versus those that live off campus. This may lend credence to the argument that a college campus can be a toxic environment and when students move off campus the constant bombardment and ease of getting food is ameliorated, thus resulting in a more favorable body composition.

Little data exists regarding weight changes in college students over the long term. However, research [13,14] in male and female college students has shown that over the Thanksgiving holiday body weight significantly increased by 0.5 kg. When students returned for follow-up testing after the holidays (i.e. Christmas and New Year's) in mid-January, on average the post holiday body weight (71.2 kg) was not significantly different from the pre-holiday weight (71.3 kg). Though this time is much shorter than the two year time span of the current study, it does provide perhaps preliminary evidence of students increasing weight initially and returning to a pre-set weight.

Two possible explanations may help explain the significant decrease in %fat with little change in body weight during the sophomore year compared to the freshman year. First, the participants were given their body weight and composition results at the completion of each visit. Therefore, participants were aware of how their body weight and composition changed during their freshman year perhaps leading to corrective changes in dietary and physical activity patterns to prevent further gain during their sophomore year. A second explanation may be related to the familiarity of their food environment coupled with body weight awareness due to participation in the study. Their freshman year represents a time where students acclimate themselves to the university's maze of eating choices (i.e. the all you can eat cafeteria and over 20 separate eating establishments). It has been shown that the unfamiliarity of food that is consumed makes it more difficult to regulate energy intake [15]. Additionally, it has been shown that the frequency of consuming restaurant food is positively associated with increased fat mass [16]. The University of Oklahoma is not unlike many college campus's today with students having the opportunity to choose from 10 restaurants located on the campus (i.e. Burger King, Wendy's, Quizno's, etc.) and perhaps more importantly are directly linked to their meal plan. As a result, when students arrive on campus as freshman the quantity of eating establishments may result in greater ease in overeating. Consequently, during their sophomore year students have had time to become familiar with food choices coupled with awareness of weight gain during their first year of college and therefore regulate their energy intake and physical activity levels to avoid weight gain. Others have shown a similar trend towards a slowing of weight gain during the sophomore year with a "corrective" loss to baseline levels (i.e. fall of freshman year) during the junior year, but no study has characterized the change and pattern of body composition during this time [5]. In fact, to our knowledge no study has directly measured body composition during the sophomore year using a true criterion method (i.e. DXA), thus other investigators have not substantiated the results seen in this study.

### Future considerations

Detailed dietary information and objective measures of physical activity were not collected; thus, the ability to make causative statements remains speculative at this time. Future studies should quantify total energy and daily macro/micro nutrient composition and objectively assess time spent in moderate to vigorous physical activity. Additionally, we would encourage future studies to measure psychosocial and behavioral issues, such as depression and anxiety as important predictors of weight gain. Lastly, we strongly urge a detailed and thorough analysis of the built environment of the college setting. Taken together, these additional considerations would allow for a pointed approach in better intervention programs whose primary objective is to curtail and staving off weight gain in a vulnerable population.

## Conclusion

In conclusion, participant's body weight increased minimally (0.2 kg) during the sophomore year of college, though a decrease in %fat (1 %fat units) was observed. In all likelihood, this observed decrease was a result of fat mass (0.6 kg) decreasing and fat-free mass (0.7 kg) increasing. Interestingly, our results found those that lived off campus saw no increase in body weight while decreasing %fat and fat mass. Relative to the freshman year, the sophomore cohort of females gained significantly less body weight while at the same time decreasing %fat and fat mass during their sophomore year.

## Competing interests

The author(s) declare that they have no competing interests.

## Authors' contributions

The study was conceived by MM, DF and MD and data collection was completed by MM, JH and HH. HH and DF analyzed the data and wrote the manuscript and MM, JH and MD revised and provided critical review of the manuscript. All authors read and approved the final manuscript.

## Pre-publication history

The pre-publication history for this paper can be accessed here:


